# Distinct TB-antigen stimulated cytokine profiles as predictive biomarkers for unfavorable treatment outcomes in pulmonary tuberculosis

**DOI:** 10.3389/fimmu.2024.1392256

**Published:** 2024-06-03

**Authors:** Arul Nancy Pandiarajan, Nathella Pavan Kumar, Nandhini Selvaraj, Shaik Fayaz Ahamed, Vijay Viswanathan, Kannan Thiruvengadam, Syed Hissar, Sivakumar Shanmugam, Ramalingam Bethunaickan, Sujatha Nott, Hardy Kornfeld, Subash Babu

**Affiliations:** ^1^ ICER India, National Institute of Allergy and Infectious Diseases (NIAID) - International Center for Excellence in Research, Chennai, India; ^2^ Department of Immunology, ICMR - National Institute for Research in Tuberculosis, Chennai, India; ^3^ Diabetology, Prof. M. Viswanathan Diabetes Research Center, Chennai, India; ^4^ Medicine, Infectious Diseases, Dignity Health, Chandler, AZ, United States; ^5^ Medicine, University of Massachusetts Medical School, Worcester, MA, United States; ^6^ Laboratory of Parasitic Diseases (LPD), National Institute of Allergy and Infectious Diseases (NIAID), National Institutes of Health (NIH), Bethesda, MD, United States

**Keywords:** tuberculosis, recurrence, TB treatment failure, cytokines, TB treatment cure

## Abstract

**Introduction:**

The assessment of tuberculosis (TB) treatment outcomes predominantly relies on sputum culture conversion status. To enhance treatment management, it is crucial to identify non-sputum-based biomarkers that can predict unfavorable outcomes. Cytokines are widely studied as diagnostic biomarkers for active TB. However, their potential as indicators for unfavorable treatment outcomes remains uncertain.

**Methodology:**

This study was conducted within a well-characterized cohort comprising newly diagnosed patients with drug-sensitive pulmonary TB, confirmed through sputum smear and culture positivity. Our objective was to elucidate the TB antigen-stimulated cytokine profile at pre-treatment and at 2 months into anti-TB treatment (ATT) in patients with unfavorable treatment outcomes (cases, *n* = 27) in comparison to recurrence-free, microbiologically cured controls (*n* = 31). Whole blood was stimulated with TB antigens using the QuantiFERON In-tube gold method, and plasma supernatants were subjected to a panel of 14 cytokine measurements.

**Results:**

In our study, pre-treatment analysis revealed that eight cytokines (IL-2, IFN-γ, TNF-α, IL-6, IL-10, IL-17A, IL-18, and GM-CSF) were significantly elevated at baseline in cases compared to cured controls, both in unstimulated conditions and following TB antigen (CFP10, ESAT6, and TB7.7) stimulation. A similar pattern was observed at the 2-month mark of ATT, with eight cytokines (IL-2, IL-10, IL-13, IFN-γ, IL-6, IL-12p70, IL-17A, and TNF-α) showing significant differences between the groups. Importantly, no variations were detected following mitogen stimulation, underscoring that these distinctive immune responses are primarily driven by TB-specific antigens.

**Conclusion:**

Our findings indicate that individuals with unfavorable TB treatment outcomes display a characteristic cytokine profile distinct from TB-cured patients, even before commencing ATT. Therefore, the levels of specific cytokine pre-treatment and at the 2-month point in the course of treatment may serve as predictive immune markers for identifying individuals at risk of unfavorable TB treatment outcomes, with these responses being predominantly influenced by TB-specific antigens.

## Introduction

Tuberculosis (TB) continues to pose a formidable global health challenge, with India contributing to a staggering 26% of the world’s new TB cases (1). The persistence of latent *Mycobacterium tuberculosis* (M.tb) infections acts as a reservoir to develop into a disease (2), and the heightened susceptibility conferred by comorbid conditions such as diabetes and HIV contributes to this burden. Furthermore, TB recurrence and treatment failure, occurring at rates of 6.8% and 4.3%, respectively, add significantly to the public health crisis (3).

Given the time-consuming and expensive nature of culture-based TB diagnosis, it is imperative to explore non-sputum-based (4, 5) biomarkers. Several studies have addressed this issue, seeking blood-based biomarkers for monitoring TB treatment response (6–8). While the World Health Organization (WHO) has revised guidelines to shorten drug-susceptible TB regimens from 6 to 4 months, this change raises concerns about increased recurrence risks (9, 10). Nonetheless, shorter treatment regimens promise better disease control and streamlined management, ultimately alleviating the functional burden on national and international TB programs (11, 12). To mitigate the risks associated with shorter treatment durations, predictive biomarkers are urgently needed to identify and stratify patients at risk of unfavorable treatment outcomes, including recurrence, treatment failure, and death. Such markers could be applied either before initiating TB treatment or, minimally, by the 2-month mark.

Cytokines, molecules involved in the immune response (13, 14), have shown promise in differentiating TB infection from active disease (15). Furthermore, specific plasma cytokines exhibit remarkable specificity in distinguishing various stages of TB, from latency to drug sensitivity and drug resistance (16, 17). However, the precise role of these cytokines in treatment outcomes remains incompletely understood. The primary objective of this study was to elucidate TB antigen-specific predictive biomarkers for unfavorable or adverse pulmonary TB (PTB) treatment outcomes. By examining these biomarkers before and during anti-TB treatment (ATT), we aim to provide valuable insights that can inform early interventions and personalized TB management strategies, ultimately enhancing the diagnosis and treatment of this pervasive infectious disease.

## Materials and methods

### Ethics statement

This study received approval from the Ethics Committee of Prof. M. Viswanathan Diabetes Research Center (ECR/51/INST/TN/2013/MVDRC/01) and the National Institute for Research in Tuberculosis (NIRT IEC No. 2014004). Informed, written consent was obtained from all participants.

### Study population

The study subjects were drawn from the EDOTS (Effect of Diabetes on Tuberculosis Severity) cohort, conducted between 2014 and 2019. Patients for this study were enrolled from a TB clinic and screened for diabetes mellitus. Inclusion criteria encompassed newly diagnosed patients with PTB with positive sputum smear and culture results. Exclusion criteria comprised individuals with prior TB episodes, those with drug-resistant TB, those undergoing TB treatment for more than a week, those taking immunosuppressive medication, those with HIV-positive status, and pregnant and lactating individuals. The diagnosis of PTB relied on positive sputum cultures grown on solid media (Lowenstein–Jensen media), with supporting chest x-ray findings. TB treatment adhered to the standards set by the Revised National Tuberculosis Control Program and was managed through government clinics in Chennai. Monthly follow-ups were conducted throughout the 6-month treatment course, with subsequent evaluations at 3-month intervals until 1 year post-treatment completion. This nested case–control study defined cases as individuals with unfavorable treatment outcomes, matched with controls who achieved recurrence-free cures by the study’s end. Cure was defined as having negative sputum cultures at both months 5 and 6 of treatment, without recurrent disease during follow-up. Treatment failure referred to positive sputum cultures at either month 5 or 6 and all were culture positive at month 2. Death included all-cause mortality during TB treatment, which includes cardiorespiratory arrest, sudden cardiac pulmonary arrest, and severe illness, and recurrence indicated an initial cure at month 6 but culture-confirmed disease recurrence before the study’s conclusion. The study included 27 cases (comprising 16 recurrences, 6 treatment failures, and 5 deaths) and 31 cured individuals, matched for age, gender, body mass index (BMI), and diabetic status. Throughout our follow-up of the cohort, we did not record any bacterial infection.

### QuantiFERON-TB gold in-tube supernatant ELISA

For TB antigen-specific immune response analysis, the third-generation QuantiFERON (QFT) plasma supernatants were employed. The QFT tubes contained three components: NIL (for immune status without stimulation), TB-Ag (with TB antigens TB 7.7, CFP10, and ESAT6), and mitogen (a positive control for the test). To collect the supernatant, 1 mL of patient whole blood was incubated in the corresponding tubes following the manufacturer’s instructions (QuantiFERON In Tube Gold Kit; Qiagen, Valencia, CA).

### Enzyme-linked immunosorbent assay

A multiplex enzyme-linked immunosorbent assay (ELISA) for cytokines was conducted to examine antigen-specific cytokines in unfavorable treatment outcomes (cases, *n* = 27) and favorable treatment outcomes (controls, *n* = 31). The assay followed the manufacturer’s instructions (R&D Systems Elisa Kit). Stimulated plasma cytokine levels were measured using Luminex Magpix with xMAP technology (Bio-Rad, Hercules, CA). The lowest detection limits for cytokines were as follows: IL-2, 9.54 pg/mL; IFN-γ, 16.6 pg/mL; TNF-α, 2.56 pg/mL; GM-CSF, 4.21 pg/mL; IL-1α, 1.88 pg/mL; IL-17A, 4.18 pg/mL; IL-5, 2.44 pg/mL; IL-10, 1.41 pg/mL; IL-13, 157.49 pg/mL; IL-18, 5.51 pg/mL; IL-1β, 4.92 pg/mL; IL-4, 5.44 pg/mL; IL-6, 1.4 pg/mL; and IL-12, 48.37 pg/mL, which were analyzed between cases and cured controls.

### Statistical analysis

All statistical analyses were conducted using GraphPad Prism Version 9. Significance between cases and controls was assessed using the Mann–Whitney test, while pre- and post-treatment comparisons within related groups were determined using the Wilcoxon test. Median and geometric mean (GM) were used to represent central tendencies, and the range (min and max) was employed to depict data distribution. The chi-square test was used to check the significance for the non-numerical data. Receiver operating characteristic (ROC) analysis was performed using R software, and Combi ROC was conducted through an online tool (http://combiroc.eu/) with the most significant variables.

## Results

### Characteristics of the study population

**Table 1** presents the demographic characteristics of the study population. There were no significant differences between the groups concerning age, gender, BMI, diabetes status, cough, smoking, alcohol consumption, or smear and culture grade.

**Table 1:** Demographics of the study population.

S. no.	Parameters	Status	Cases (*n* = 27)	Controls (*n* = 31)	Significance
1	**Age**	in years	44 (23 to 65)	50 (25 to 60)	0.837
2	**Gender**	Female	3 (11.1%)	5 (16.1%)	0.580
		Male	24 (88.8%)	26 (83.9%)	
3	**BMI**		16.89 (13.27 to 25.11)	18.36 (12.74 to 26.14)	0.990
4	**Diabetes status**				0.407
		Non-diabetes	11 (56.25%)	16 (30.7%)	
		Diabetes	16 (43.75%)	15 (69.2%)	
5	**HbA1C**	mmol/mol	4.9 to 12.8 (6.5)	5 to 13.3 (6.50)	0.978 (0.65) **^#^**
6	**Cough**				0.279
		Absence	1 (3.7%)	0 (0%)	
		Presence	26 (96.3%)	31 (100%)	
7	**Dyslipidemia**				NA
		Absence	27 (100%)	31 (100%)	
		Presence	0 (0%)	0 (0%)	
8	**Smoking**				0.450
		Never	12 (44.4%)	16 (51.6%)	
		Past	4 (14.8%)	7 (22.6%)	
		Current	11 (40.7%)	8 (25.8%)	
9	**Alcohol**				0.701
		Never	8 (29.6%)	12 (38.7%)	
		Past	4 (14.8%)	3 (9.7%)	
		Current	15 (55.5%)	16 (51.6%)	
10	**Cavity**				0.062
		Absence	20 (74%)	15 (48.4%)	
		Presence	6 (22.22%)	9 (29.0%)	
		Not known	1 (3.7%)	7 (22.6%)	
11	**Smear**				0.091
		1+	16 (59.3%)	26 (83.9%)	
		2+	10 (37.0%)	5 (16.1%)	
		3+	1 (3.7%)	0 (0%)	
12	**Culture**				0.178
		1+	10 (37.0%)	19 (61.3%)	
		2+	5 (18.5%)	4 (12.9%)	
		3+	12 (44.4%)	8 (25.8%)	
13	**Chest x-ray**	CXR score	24 (7 to 90)	39.5 (4 to 105)	0.73
14	**Clinical parameters**				
	**WBC absolute count**	Absolute count	8,200 to 19,900 (12,100)	5,500 to 17,800 (9,900)	0.02
	**Lymphocyte**	Absolute count	432 to 2,751 (1,782)	760 to 5,162 (1,952)	0.063
	**Monocyte**	Absolute count	111 to 2,610 (688)	64 to 1,414 (484)	0.036
	**Platelet**	Microliter	142 to 661 (414)	169 to 687 (432)	0.929

Numerical values are presented as n (%) and in median (range). Significance between the groups was achieved by Mann–Whittney test, **^#^** Independent t-test and chi-square test (for non-numerical data).

### Elevated cytokines in unstimulated plasma of cases before and at month 2 of anti-TB treatment

**Figure 1:**
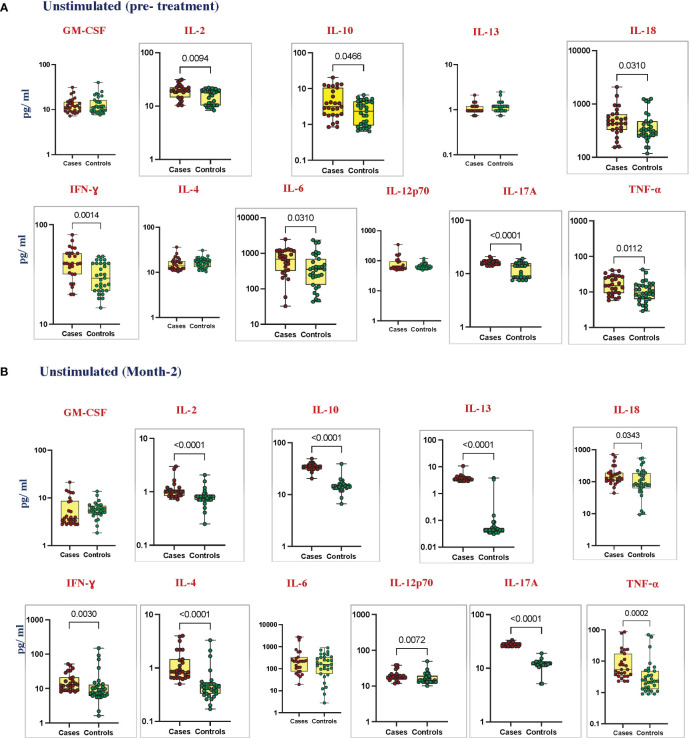
Elevated cytokines in unstimulated plasma can predict unfavorable treatment outcomes before treatment (baseline). Unstimulated plasma levels of cytokines were measured in cases (*n* = 27) and controls (*n* = 31) **(A)** pre-treatment and **(B)** after 2 months of TB treatment. These data were represented in a box-and-whiskers plot where each dot represents every single participant in its group. The median line was presented at the middle of the box with its error bar at both ends. Mann–Whitney test was used to calculate the *p*-values of the unrelated groups. Grey boxes refer to the cytokines that were significantly different.

To estimate the unstimulated cytokine levels in cases and controls, we measured the levels of GM-CSF, IL-2, IL-10, IL-13, IL-18, IFN-γ, IL-1α, IL-4, IL-6, IL-12p70, IL-17A, and TNF-α at pre-treatment (**Figure 1A**); IL-1α, IL-5, and IL-1β (**Supplementary Figure 2A**) at month 2 of ATT (**Figure 1B**); and IL-1α, IL-5, and IL-1β (**Supplementary Figure 2B**). Unstimulated plasma levels of IL-2 (*p* = 0.0094, GM of 18.72 pg/mL in cases vs. 14.89 pg/mL in controls), IL-10 (*p* = 0.0466, GM of 3.74 pg/mL in cases vs. 2.086 pg/mL in controls), IL-18 (*p* = 0.0310, GM of 463.8 pg/mL in cases vs. 354.2 pg/mL in controls), IFN-γ (*p* = 0.0014, GM of 40.42 pg/mL in cases vs. 29.32 pg/mL in controls), IL-6 (*p* = 0.0310, GM of 545.1 pg/mL in cases vs. 314.8 pg/mL in controls), and TNF-α (*p* = 0.0112, GM of 15.57 pg/mL in cases vs. 9.875 pg/mL in controls) were significantly higher in cases than in controls before treatment. At month 2 of treatment, unstimulated plasma levels of IL-2 (*p* < 0.0001, GM of 1.055 pg/mL in cases vs. 0.7781 pg/mL in controls), IL-10 (*p* < 0.0001, GM of 34.47 pg/mL in cases vs. 13.06 pg/mL in controls), IL-13 (*p* < 0.0001, GM of 3.696 pg/mL in cases vs. 0.060 pg/mL in controls), IL-18 (*p* = 0.0343, GM of 153.7 pg/mL in cases vs. 65.87 pg/mL in controls), IFN-γ (*p* = 0.0030, GM of 15.09 pg/mL in cases vs. 10.23 pg/mL in controls), IL-4 (*p* < 0.0001, GM of 1.076 pg/mL in cases vs. 0.463 pg/mL in controls), IL-12p70 (*p* = 0.0072, GM of 19.05 pg/mL in cases vs. 16.22 pg/mL in controls), IL-17A (*p* < 0.0001, GM of 27.95 pg/mL in cases vs. 10.62 pg/mL in controls), and TNF-α (*p* = 0.0002, GM of 7.387 pg/mL in cases vs. 3.015 pg/mL in controls) were significantly higher in cases than in controls. There were no significant differences in GM-CSF, IL-1α, IL-5, IL-13, IL1-β, IL-4, and IL-12p70 at baseline and GM-CSF, IL1-α, IL-5, IL1-β and IL-6 at month 2 between cases and controls.

### Elevated cytokines in TB antigen-stimulated plasma of cases before and at month 2 of anti-TB treatment

**Figure 2:**
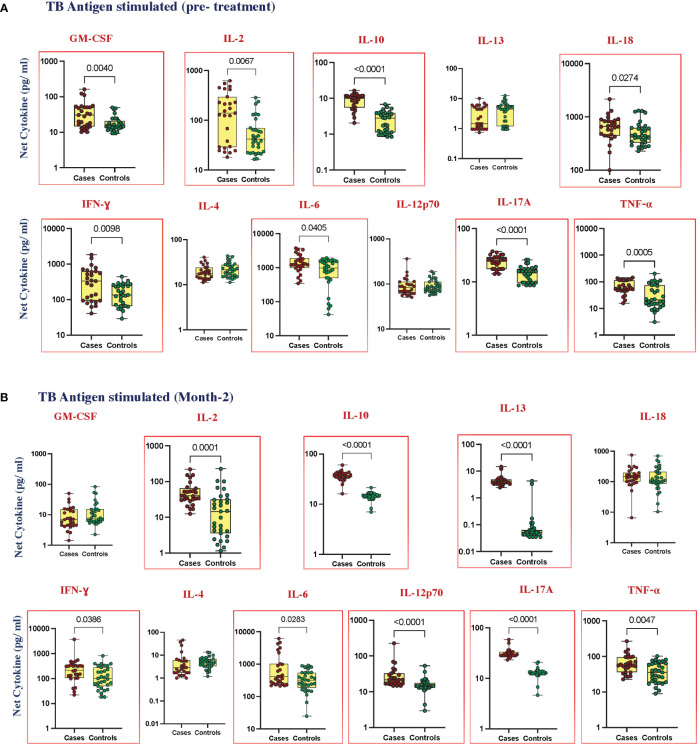
Elevated cytokines in TB antigen-stimulated plasma can predict unfavorable treatment outcomes **(A)** before treatment (baseline) **(B)** after treatment at month 2. TB antigen-stimulated plasma levels of cytokines were measured in cases (*n* = 27) and controls (*n* = 31) before treatment. This data was represented in a box-and-whiskers plot where each dot represents every single participant in its groups. The median line was presented at the middle of the box with its error bar at both ends. The Mann–Whitney test was used to calculate the *p*-values of the unrelated groups. Red boxes refer to the cytokines that were significantly different.

To elucidate the TB antigen-stimulated cytokine levels in cases and controls, we estimated the production of GM-CSF, IL-2, IL-10, IL-13, IL-18, IFN-γ, IL-4, IL-6, IL-12p70, IL-17A, and TNF-α at pre-treatment (**Figure 2A**); IL-1α, IL-5, and IL-1β (**Supplementary Figure 2C**) at month 2 of ATT (**Figure 2B**); and IL-1α, IL-5, and IL-1β (**Supplementary Figure 2D**). Upon TB antigen stimulation, the plasma levels of GM-CSF (*p* = 0.0040, GM of 29.75 pg/mL in cases vs. 16.92 pg/mL in controls), IL-2 (*p* = 0.0067, GM of 112.2 pg/mL in cases vs. 48.15 pg/mL in controls), IL-10 (*p* < 0.0001, GM of 8.209 pg/mL in cases vs. 2.202 pg/mL in controls), IL-18 (*p* = 0.0274, GM of 565.3 pg/mL in cases vs. 486.8 pg/mL in controls), IFN-γ (*p* = 0.0098, GM of 266.0 pg/mL in cases vs. 135.2 pg/mL in controls), IL-6 (*p* = 0.0405, GM of 1322 pg/mL in cases vs. 694.7 pg/mL in controls), IL-17A (*p* < 0.0001, GM of 23.48 pg/mL in cases vs. 14.06 pg/mL in controls), and TNF-α (*p* = 0.0005, GM of 62.94 in cases vs. 27.06 pg/mL in controls) were significantly higher in cases than in cured controls at pre-treatment. At month 2, TB antigen-stimulated plasma levels of IL-2 (*p* = 0.0001, GM of 44.67 pg/mL in cases vs. 11.65 pg/mL in controls), IL-10 (p, 0.0001, GM of 36.32 pg/mL in cases vs. 14.27 pg/mL in controls), IL-13 (*p* < 0.0001, GM of 4.243 pg/mL in cases vs. 0.047 pg/mL in controls), IFN-γ (*p* = 0.0386, GM of 195.9 pg/mL in cases vs. 106.9 pg/mL in controls), IL-6 (*p* = 0.283, GM of 613.7 pg/mL in cases vs. 291.9 pg/mL in controls), IL-12p70 (*p* < 0.0001, GM of 25.84 pg/mL in cases vs. 14.85 pg/mL in controls), IL-17A (*p* < 0.0001, GM of 30.72 pg/mL in cases vs. 12.58 pg/mL in controls), and TNF-α (*p* = 0.0047, GM of 60.73 pg/mL in cases vs. 34.38 pg/mL in controls) were elevated in cases compared to controls. There was no significant difference in IL-1α, IL-5, IL-13, IL-1β, IL-4, and IL-12p70 at baseline and GM-CSF, IL-1α, IL-5, IL-18, IL-1β, and IL-4 at month 2 between cases and controls.

### No change in cytokine level in mitogen-stimulated plasma

To elucidate the mitogen-stimulated cytokine levels in cases and controls, we estimated the production of GM-CSF, IL-1α, IL-2, IL-5, IL-10, IL-13, IL-18, IFN-γ, IL-1β, IL-4, IL-6, IL-12p70, IL-17A, and TNF-α at pre-treatment (**Supplementary Figure 1A**) and at month 2 of ATT (**Supplementary Figure 1B**). There were no significant differences in the cytokine levels between cases and cured controls in pre-treatment as well as at month 2 after treatment.

### ROC analysis reveals that the pre-treatment and month 2 levels of cytokines can serve as predictive biomarkers for unfavorable treatment outcomes

To assess the potential of unstimulated and TB antigen-stimulated cytokine levels as biomarkers for unfavorable treatment outcomes, we conducted ROC analysis to evaluate sensitivity and specificity. **Table 2** demonstrates the ROC analysis results for IL-10, IL-17A, and TNF-α upon TB antigen stimulation before treatment, showing good AUC values of 0.85, 0.86, and 0.76, respectively. Sensitivity ranged from 63% to 93%, and specificity varied from 58% to 100%. After treatment at month 2, cytokines such as IL-2, IL-10, and IL-17A exhibited high AUC values (0.78, 0.99, and 1) with corresponding sensitivity (81%, 96%, and 100%) and specificity (71%, 100%, and 100%) in distinguishing cases and controls (as shown in **Table 3**). Similarly, in the unstimulated condition, IL-17A alone demonstrated a good AUC of 1.0 with 100% sensitivity and specificity before treatment. At month 2, IL-10, IL-17A, and TNF-α presented high AUC values (0.98, 1, and 0.77) with sensitivity (100%, 100%, and 88.9%) and specificity (96.7%, 100%, and 61%) in differentiating cases and controls.

**Table 2:** ROC analysis for cytokines before treatment.

Cytokines	Unstimulated				TB Antigen stimulated				Mitogen stimulated			
	Sensitivity	Specificity	AUC	*p*-value	Sensitivity	Specificity	AUC	*p*-value	Sensitivity	Specificity	AUC	*p*-value
**GM-CSF**	48.15	64.52	0.503	0.963	55.56	87.1	0.717	**0.002**	40.74	74.19	0.522	0.786
**IL-2**	62.96	70.97	0.697	**0.004**	62.96	80.65	0.705	**0.004**	29.63	93.55	0.529	0.721
**IL-10**	37.04	100	0.651	**0.045**	62.96	100	**0.853**	**<0.001**	66.67	51.61	0.545	0.564
**IL-18**	77.78	58.06	0.665	**0.027**	59.26	77.42	0.668	**0.023**	29.63	90.32	0.535	0.658
**IFN-γ**	81.48	61.29	0.74	**<0.001**	55.56	90.32	0.697	**0.008**	40.74	87.1	0.527	0.741
**IL-6**	70.37	67.74	0.665	**0.025**	81.48	48.39	0.657	**0.031**	60	61.29	0.575	0.343
**IL-17A**	88.89	67.74	**0.795**	**<0.001**	88.89	70.97	**0.864**	**<0.001**	59.26	58.06	0.517	0.826
**TN**F-α	40.74	93.55	0.693	**0.006**	92.59	58.06	**0.762**	**<0.001**	84.62	32.26	0.511	0.886

Receiver operating characteristic (ROC) analysis represents sensitivity, specificity, area under the curve (AUC), and the *p*-value for significant cytokines between cases and controls in unstimulated, and TB antigen- and mitogen-stimulated conditions before TB treatment. Significant AUC and *p*-values are in boldface.

**Table 3:** ROC analysis for cytokines after treatment at month 2.

Cytokines	Unstimulated				TB antigen stimulated				Mitogen stimulated			
	Sensitivity	Specificity	AUC	*p*-value	Sensitivity	Specificity	AUC	*p*-value	Sensitivity	Specificity	AUC	*p*-value
**GM-CSF**	62.96	87.1	0.628	0.129	37.04	96.77	0.614	0.143	77.78	35.48	0.511	0.89
**IL-2**	62.96	87.1	**0.809**	**<0.001**	81.48	70.97	**0.784**	**<0.001**	51.85	61.29	0.527	0.732
**IL-10**	100	96.77	**0.975**	**<0.001**	96.3	100	**0.99**	**<0.001**	70.37	45.16	0.526	0.743
**IL-18**	88.89	51.61	0.662	0.028	66.67	54.84	0.573	0.34	18.52	67.74	0.501	0.994
**IFN-γ**	100	51.61	0.724	**0.001**	85.19	48.39	0.658	0.031	25.93	100	0.563	0.429
**IL-6**	59.26	58.06	0.558	0.452	33.33	96.77	0.668	0.019	55.56	67.74	0.557	0.477
**IL-17A**	100	100	**1**	**<0.001**	100	100	**1**	**<0.001**	77.78	3.23	0.521	0.794
**TN**F-α	88.89	61.29	**0.773**	**<0.001**	74.07	61.29	0.714	**0.001**	88.89	38.71	0.554	0.488

Receiver operating characteristic (ROC) analysis represents sensitivity, specificity, area under the curve (AUC), and the *p*-value for significant cytokines between cases and controls in unstimulated, and TB antigen- and mitogen-stimulated conditions after TB treatment at month 2. Significant AUC and *p*-values are in boldface.

### Altered cytokine levels at pre- and post-treatment between cases and controls under unstimulated and TB antigen-stimulated conditions

**Figure 3:**
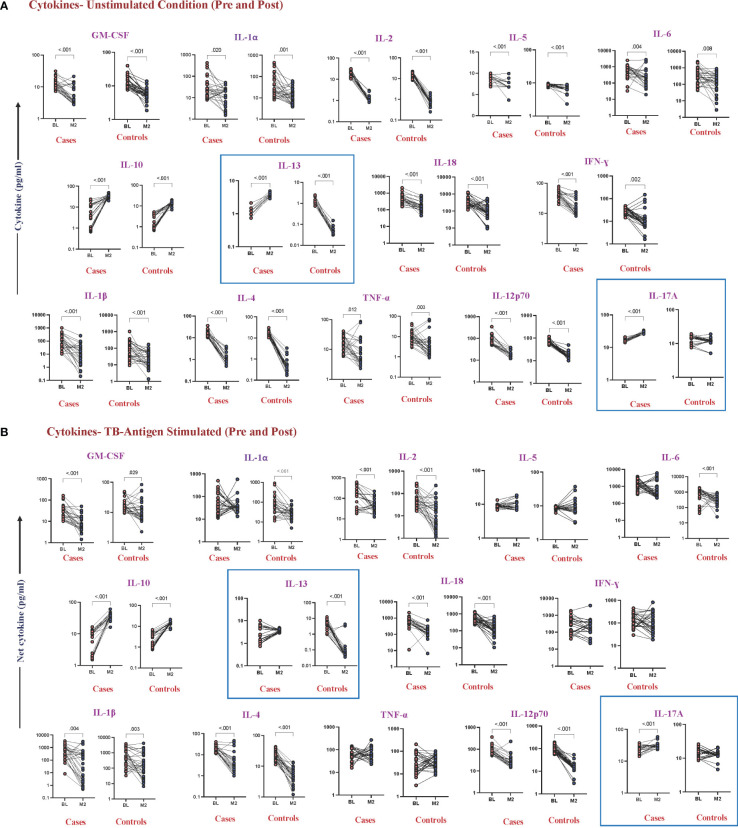
Altered cytokine levels in unstimulated and TB antigen-stimulated plasma were observed in cases and controls before and after 2 months of TB treatment. Wilcoxon rank analysis was performed to determine the *p*-value in these related groups. **(A)** Unstimulated. The blue box represents elevated levels of cytokines at month 2 in cases. **(B)** TB antigens stimulated. The blue box represents altered levels of cytokines between cases and controls in both pre- and post-TB treatment.

To examine whether there were any alterations in cytokine levels before and after ATT at month 2 between cases and controls, we found that IL-13 (cases, *p* < 0.001, GM of 1.05 pg/mL in BL vs. 3.541 pg/mL in M2) (controls, *p* < 0.001, GM of 1.151 pg/mL in BL vs. 0.045 pg/mL in M2) and IL-17A (cases, *p* < 0.001, GM of 16.25 pg/mL in BL vs. 27.94 pg/mL in M2) (controls, *p* = 0.430, GM of 12.01 pg/mL in BL vs. 11.64 pg/mL in M2) were significantly increased in cases at month 2 in the unstimulated condition (**Figure 3A**). The levels of IL-13 (cases, *p* = 0.337, GM of 2.25 pg/mL in BL vs. 4.04 pg/mL in M2) (controls, *p* < 0.001, GM of 3.36 pg/mL in BL vs. 0.07 pg/mL in M2) and IL-17A (cases, *p* < 0.001, GM of 23.47 pg/mL in BL vs. 30.72 pg/mL of M2) (controls, *p* = 0.129, GM of 14.05 pg/mL in BL vs. 12.75 pg/mL in M2) continue to be different under TB antigen-stimulated conditions (**Figure 3B**). Levels of cytokines upon mitogen stimulation did not show significant differences between cases and controls, although they underwent changes pre- and post-treatment (**Supplementary Figure 1C**).

### Combinatorial ROC analysis revealed the best cytokine combinations as predictive biomarkers for unfavorable treatment outcomes at pre-treatment and at month 2

**Figure 4:**
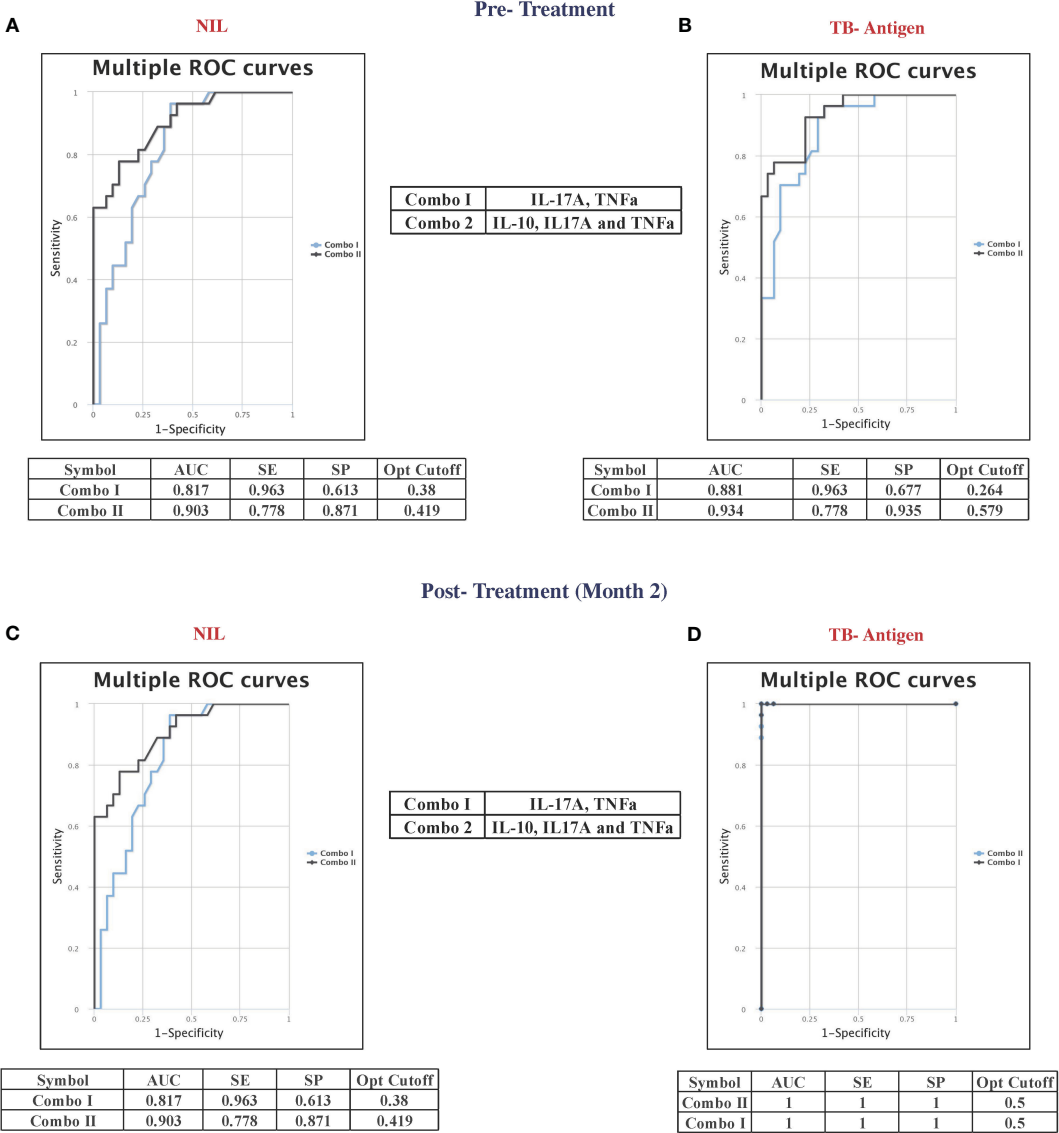
Multiple combinations of cytokines as a predictive biomarker discriminate unfavorable treatment outcomes from cured controls before treatment (baseline) [**(A)** NIL and **(B)** TB antigens] and after 2 months of treatment [**(C)** NIL and **(D)** TB antigens]. The curve was represented in a combination of receiver’s operator characteristic (ROC). AUC, area under the curve; SE, sensitivity; SP, specificity.

Combined ROC analysis was conducted to evaluate the efficiency of cytokine combinations before treatment (shown in **Figure 4A**—NIL and **Figure 4B**—TB antigen) and after treatment at month 2 (shown in **Figure 4C**—NIL and **Figure 4D**—TB antigen). Cytokine signatures for combi-ROC included IL-17A and TNF-α as combo I and IL-10, IL-17A, and TNF-α as combo II. Combo I and II exhibited AUC values of 0.8 and 0.9 with a sensitivity of 96% and 78% and a specificity of 61% and 87%, respectively, in the unstimulated condition (NIL). Under TB antigen-stimulated conditions, AUC values of combo I and II showed 0.8 and 0.9 with a sensitivity of 96% and 78% and a specificity of 68% and 94% before treatment. At month 2 after treatment, AUC values of combo I and II were 0.8 and 0.9 with sensitivity (96% and 78%) and specificity (61% and 87%) in the unstimulated condition, whereas under TB antigen-stimulated conditions, AUC values of combo I and II showed 1 with 100% sensitivity and specificity. Thus, certain cytokine levels in unstimulated and TB antigen stimulation are highly useful in the early prediction of unfavorable treatment outcomes in patients with PTB.

## Discussion

TB remains a major global health concern, particularly in countries like India, which bears a substantial burden of the disease. The high rates of TB recurrence and treatment failure, coupled with the lack of reliable biomarkers to predict treatment outcomes (17), emphasize the critical need for better tools to guide TB management. This study aimed to investigate cytokines as potential predictive biomarkers for unfavorable TB treatment outcomes, specifically focusing on their response to TB antigens before and during the early phase of ATT.

Our findings revealed that individuals with unfavorable treatment outcomes exhibited distinct cytokine profiles, characterized by heightened levels of several cytokines, both before the initiation of treatment and at the 2-month mark during anti-TB therapy (18, 19). Notably, these differences were particularly pronounced when analyzing the immune responses to TB antigens, such as CFP10, ESAT6, and TB7.7, and were not observed with mitogen stimulation. This suggests that the immune responses driving these observed differences are primarily triggered by the TB infection itself.

Comparing our results to previous studies (20–22), it is evident that altered cytokine levels have been consistently associated with adverse TB treatment outcomes and also with disease severity (19, 23–26). While some studies have focused on baseline cytokine profiles, our investigation extends to the early phase of treatment, providing valuable insights into dynamic changes during therapy. For example, IFN-γ and TNF-α levels have previously been linked to relapse cases (27), but our study demonstrates their elevation both before and during early treatment in individuals with unfavorable outcomes. Importantly, our research highlights that the observed cytokine patterns were not significantly affected by comorbid conditions like diabetes, age, and BMI, underlining their potential as robust predictors of TB treatment response. This aspect is particularly promising for clinical application, as these biomarkers appear to be reliable indicators even in the presence of confounding factors. TB risk factors like low BMI have substantially influenced the cytokine and chemokine levels in both active and latent TB (28, 29).

Our ROC data clearly indicate that IL-10, IL-17A, and TNF-α could serve as possible predictive biomarkers for adverse treatment outcomes even before the start of TB treatment. The combination of IL-17A and TNF-α, with or without the inclusion of IL-10, had an AUC of 1 and 100% sensitivity and specificity in differentiating adverse treatment outcomes from cured individuals. Certain plasma cytokines, such as IL-4, IL-5, IL-10, IL-13, and IL-37, were altered following ATT when compared with latent and healthy individuals (30). Moreover, several other studies showed altered cytokine levels in whole blood plasma from QuantiFERON-TB Gold plus before and after 2 months of TB treatment, which was described to be helpful in assessing the efficiency of ATT (31, 32), as well as the TB treatment response. Although interferon-gamma release withdraws its ability to diagnose disease progression from latency (33, 34), it is essential to analyze its potential in differentiating two extreme treatment outcomes like recurrence and cure. Multiple studies have shown the importance of cytokines in TB disease and treatment outcomes, which are influenced by comorbid conditions like diabetes and HIV (35–37) to a great extent. Additionally, studies have also shown that IL-13 and IL-5 declined in patients who experienced treatment failure (38). In contrast to these results, our results show no significant difference in IL-13 and IL-5 before treatment in adverse TB treatment outcomes. Apart from this, the late reduction of IL-6 plasma level was found to be a promising marker for slow TB treatment responders when compared with fast responders after 6 weeks of ATT (39). These cytokines not only govern the overall efficiency of TB treatment but also help in treatment monitoring. In this study, IL-13 was found to be unaltered between cases and controls before TB treatment but elevated drastically in cases and declined in controls after 2 months of TB treatment when compared with before treatment in the unstimulated condition. Upon TB antigen stimulation, though no significance was observed between pre- and post-levels of IL-13 in cases, controls were marked by their reduced level after 2 months of TB treatment.

While our previous studies have explored various biomarkers for TB treatment outcomes, including chemokines, matrix metalloproteinases (MMPs), tissue inhibitors of matrix metalloproteinases (TIMPs), acute phase proteins (APPs), microbial translocation markers, chitinase, and indoleamine 2,3-dioxygenase (IDO) (40–42), our current work specifically focuses on TB antigen-stimulated cytokines. This emphasis is essential because cytokines play a crucial role in immune responses during TB infection and treatment. Our findings suggest that cytokines hold a great promise as predictive markers, alongside other markers like CXCR3, CXCL9, CXCL10, CXCL11, and TNF-α, which have been implicated in TB diagnosis, treatment monitoring, and outcomes (43–45).

In summary, our study results suggest that measuring IL-17A and TNF-α in QFT supernatants at month 2 of TB treatment accurately discriminates between individuals destined for cure vs. adverse treatment outcomes. Our results further show that only an M.tb antigen-specific response offers predictive efficacy, and that this can conveniently be tested using QFT supernatants. Limitations of our study include the relatively small sample size, the preliminary nature of the study design, and a case–control ratio of only approximately 1. Nonetheless, our findings merit confirmation in larger cohorts at multiple sites, potentially using this test result to stratify patients into those who are likely to achieve cure with 4 months of TB treatment vs. those who will require a longer duration of treatment. These findings offer a potential breakthrough in the field of TB management, enabling early identification of individuals at risk for poor treatment responses. Further research and validation studies are warranted to confirm the utility of these cytokine biomarkers in clinical practice and to explore their role in guiding personalized TB treatment strategies. Ultimately, improving our ability to predict treatment outcomes will help mitigate the burden of TB and enhance the effectiveness of TB control programs worldwide.

## Data availability statement

The original contributions presented in the study are included in the article/**Supplementary Material**. Further inquiries can be directed to the corresponding author.

## Ethics statement

The studies involving humans were approved by Prof. M. Viswanathan Diabetes Research Center and the National Institute for Research in Tuberculosis. The studies were conducted in accordance with the local legislation and institutional requirements. The participants provided their written informed consent to participate in this study.

## Author contributions

AP: Validation, Writing – review & editing, Data curation, Formal analysis, Methodology, Software. NK: Conceptualization, Data curation, Formal analysis, Supervision, Validation, Writing – original draft, Writing – review & editing. NS: Methodology, Writing – review & editing. SA: Formal analysis, Software, Writing – review & editing. VV: Funding acquisition, Project administration, Resources, Writing – review & editing. KT: Data curation, Formal analysis, Software, Writing – review & editing. SH: Project administration, Resources, Writing – review & editing. SS: Data curation, Methodology, Supervision, Writing – review & editing. RB: Methodology, Resources, Supervision, Writing – review & editing. SN: Data curation, Validation, Writing – review & editing. HK: Writing – review & editing, Conceptualization, Data curation, Funding acquisition, Resources. SB: Writing – review & editing, Conceptualization, Data curation, Funding acquisition, Project administration, Resources, Supervision, Visualization, Writing – original draft.

## Supplementary material

Supplementary Figure 1 Cytokine levels in mitogen-stimulated plasma between favorable and unfavorable treatment outcomes **(A)** before treatment and **(B)** after treatment at month 2. Mitogen-stimulated plasma levels of cytokines were measured in cases (*n* = 27) and controls (*n* = 31) before TB treatment. These data were represented in a box-and-whiskers plot where each dot represents every single participant in its group. The median line was presented at the middle of the box with its error bar at both ends. The Mann–Whitney test was used to calculate the *p*-values of the unrelated groups. No significant difference in cytokine levels upon mitogen stimulation before TB treatment and after ATT at month 2. **(C)** Cytokine levels in mitogen-stimulated plasma in cases and controls before and after 2 months of TB treatment. Wilcoxon rank analysis was performed to determine the *p*-value in these related groups. A similar trend of cytokines in both groups in pre- and post-TB treatment represents the non-influential pattern upon mitogen stimulation.

Supplementary Figure 2 Cytokine levels in unstimulated plasma [**(A)** before treatment and **(B)** after treatment at month 2] and TB antigen-stimulated plasma [**(C)** before treatment and **(D)** after treatment at month 2] between favorable and unfavorable treatment. These are the common cytokines (IL-1α, IL-5, and IL-1β), which are not significant between the groups in both unstimulated and TB antigen-stimulated conditions.
