# Supplementary figures and images for: Distinct TB-antigen stimulated cytokine profiles as predictive biomarkers for unfavorable treatment outcomes in pulmonary tuberculosis
Source: Front Immunol. 2024 Jun 3;15:1392256. doi: 10.3389/fimmu.2024.1392256 (PMC11180841; doi:10.3389/fimmu.2024.1392256)

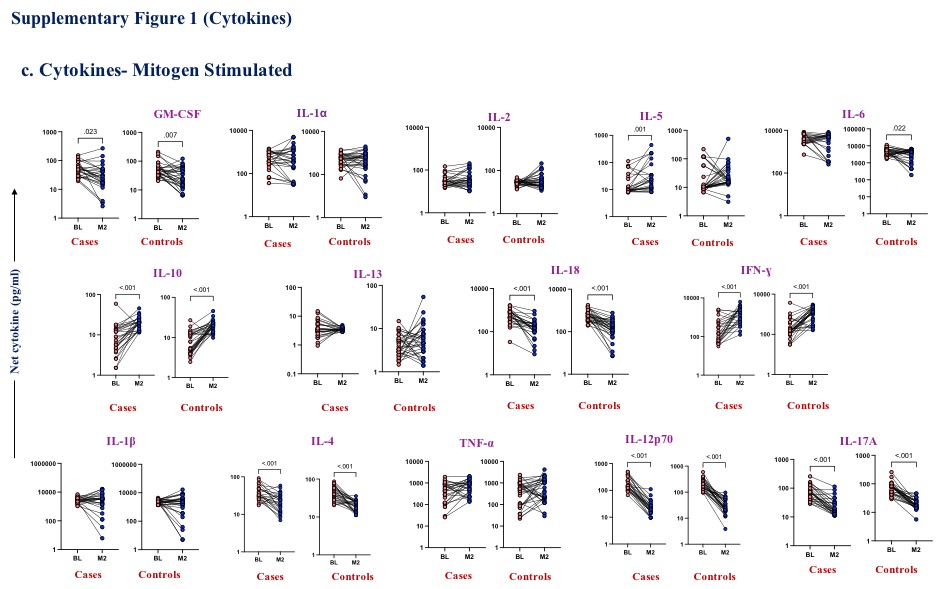

Supplement: Supplementary Figure 1 — Cytokine levels in mitogen-stimulated plasma between favorable and unfavorable treatment outcomes (A) before treatment and (B) after treatment at month 2. Mitogen-stimulated plasma levels of cytokines were measured in cases (n = 27) and controls (n = 31) before TB treatment. These data were represented in a box-and-whiskers plot where each dot represents every single participant in its group. The median line was presented at the middle of the box with its error bar at both ends. The Mann–Whitney test was used to calculate the p-values of the unrelated groups. No significant difference in cytokine levels upon mitogen stimulation before TB treatment and after ATT at month 2. (C) Cytokine levels in mitogen-stimulated plasma in cases and controls before and after 2 months of TB treatment. Wilcoxon rank analysis was performed to determine the p-value in these related groups. A similar trend of cytokines in both groups in pre- and post-TB treatment represents the non-influential pattern upon mitogen stimulation. [file Image_1.jpg]

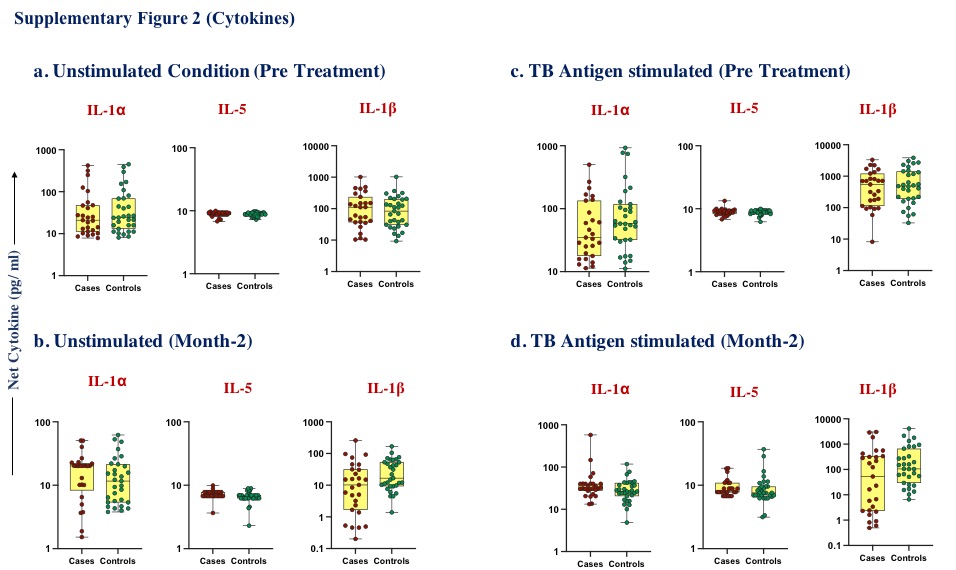

Supplement: Supplementary Figure 2 — Cytokine levels in unstimulated plasma [(A) before treatment and (B) after treatment at month 2] and TB antigen-stimulated plasma [(C) before treatment and (D) after treatment at month 2] between favorable and unfavorable treatment. These are the common cytokines (IL-1α, IL-5, and IL-1β), which are not significant between the groups in both unstimulated and TB antigen-stimulated conditions. [file Image_2.jpg]
